# Supplementary material for: Are carbon emissions trading and green financial instruments synergistic? -Comprehensive quantitative research based on content analysis
Source: PLoS One. 2024 Mar 7;19(3):e0298601. doi: 10.1371/journal.pone.0298601 (PMC10919601; doi:10.1371/journal.pone.0298601)
Supplement: S1 Data — (ZIP) [file pone.0298601.s001.zip › Data/地级市Z_F工作报告.zip!/╣π╬≈/╙±┴╓╩╨/└┤╫╘╣1⁄2╓┌║┼í╛┬φ┐╦╩2╛▌═°í┐_2016.docx]

各位代表：

现在，我代表市人民政府向大会作政府工作报告，请予审议，并请市政协委员和列席会议的同志提出意见。

一、2015年和“十二五”工作回顾

2015年是2008年国际金融危机以来经济形势最为严峻的一年。面对前所未有的困难和挑战，我们坚决贯彻落实中央、自治区和市委的决策部署，统筹做好稳增长、促改革、调结构、惠民生、防风险各项工作，努力化解经济下行压力，经济社会发展保持了稳中有进、稳中向好的良好态势。

经济实现稳定增长。制定45条稳增长措施、工业稳增长八大专项行动、市领导联系服务重点企业等一系列“定向精准”的政策举措，全面打好稳增长“组合拳”，主要经济指标平稳较快增长。初步统计，全市生产总值增长8.9%，财政收入增长8.9%，固定资产投资增长18.5%，规模以上工业增加值增长11.3%，社会消费品零售总额增长10%，城乡居民收入分别增长8.1%、10.5%。

产业发展提质增效。完成工业投资860 亿元，55个工业项目竣工投产，新增规模以上工业企业16 家，120 多个新产品获得国家、自治区认证。完成商贸物流项目投资11.3亿元，中鼎电商城、银丰国际中药港等电子商务平台和结算平台建设加快推进，新增限额以上贸易企业27家、外贸企业52家。“五彩田园”、大容山国家森林公园被评为国家4A级景区。新增农民合作社476个、家庭农场483个。

项目建设强力推进。深入开展重大项目攻坚，突出抓好47 个自治区统筹项目、550个市统筹项目、58个重点攻坚项目、36个市领导联系项目和55 个计划竣工产业项目，新开工重大项目146个，竣工58个，完成投资515亿元。实施市政项目285个，完成中心城区9大公园绿地建设工程、10条主干道绿化景观改造工程、100条小街小巷绿化补植工程，城市面貌焕然一新。改革开放成效凸显。深化农村综合改革，农村产权抵押贷款余额7.5亿元，融资担保18.2亿元。推进行政审批流程再造，固定资产投资项目审批由270多天减少到50多天。全面启动“三证合一”“先照后证”“一照一码”工作，新增市场主体2.9万户。深化投融资改革，15家企业在区域性股权交易市场挂牌。引进项目63 个，其中10 亿元以上项目17个。

民生事业加快发展。按照社会政策要托底的要求，切实抓好民生工作。完成民生支出226.4亿元，增长26.9%。投入扶贫资金1.2亿元，减少农村贫困人口6.5万人。全面完成为民办实事十项工程。新增就业8.5万人。完成各类保障房投资11.8亿元，增长48.6%，开工建设保障房1 万套，建成4222套。随着2015年主要目标任务的完成，我市“十二五”胜利收官。“十二五”时期，是我市经受严峻考验、实现跨越发展的五年。面对复杂多变的国内外形势、持续加大的经济下行压力和接踵而来的困难挑战，全市人民在自治区党委、政府和市委的坚强领导下，团结拼搏，砥砺奋进，攻坚克难，矢志不渝把改革开放和现代化建设事业推向前进，全市面貌发生新的历史性变化。玉林的发展，站到了新的更高起点上。

这是经济保持平稳较快增长、综合实力迈上新台阶的五年。我们坚持发展第一要务，坚持稳中求进的工作总基调，主动适应经济发展新常态，积极破解资金、土地、资源等各种瓶颈制约，全面加快经济结构调整和发展方式转变，大力培育新的经济增长点，努力提高发展质量和效益，全市经济持续健康发展。地区生产总值由2010 年的840.3亿元增加到2015年的1450 亿元，年均增长9.8%；财政收入由69 亿元增加到139.6 亿元，年均增长15.1%；全社会固定资产投资由615.6亿元增加到1364亿元，年均增长24.8%；规模以上工业增加值由209.5 亿元增加到464.2 亿元，年均增长15.3%。生产总值相当于2010年的1.6倍，全社会固定资产投资翻1.6 番，财政收入和规模以上工业增加值翻1番以上。人均地区生产总值达2.6万元，折合约4000美元，经济发展总体进入中等收入阶段。

这是产业转型升级有力推进、结构调整取得新突破的五年。我们坚持产业优先发展，深入实施工业跨越发展“1131工程”和“十大行动计划”，大力推进商贸兴市，实施现代农业“三个100”示范工程，推动三次产业融合发展。八大园区、八大产业发展提速，年产值超百亿元园区达到7个，超百亿元产业达到5个。玉柴铸造中心、玉柴气体发动机、中金50万吨精制镍铁及120万吨镍合金深加工等重大项目建成投产，新增规模以上工业企业449家、亿元企业300家。玉柴车用柴油机市场占有率跃居全国第一，龙潭进口再生资源园区获批为国家“城市矿产”示范基地。新增高新技术企业17家，创建企业技术中心、工程院等科研机构85家，自主研发欧Ⅵ柴油机等国内领先的新产品，粉垄深耕深松技术获中国产学研合作创新成果一等奖。毅德、大润发、富安居、汽车新城等重大商贸项目建成开业，荣获“中国南方药都”称号。建成2个4A级景区、9个3A级景区、6 个4星级以上乡村旅游区，旅游总收入年均增长30.3%。建成50亩以上连片种植基地2216个、无公害农产品示范基地131个，新增农业龙头企业121家，广西“五彩田园”现代特色农业（核心）示范区被认定为国家级农业产业化示范基地，获“中国农业公园”称号。三次产业结构由20.4：44.5：35.1 调整优化为17.8：44.1：38.1，工业化率由1.9提高到2.1，进入工业化中期阶段。

这是基础设施建设全面发力、发展能力得到新提升的五年。针对我市基础设施特别是交通设施落后的突出瓶颈，我们突出抓好一批打基础、利长远的项目，全面夯实发展基础。建设投资亿元以上项目1672个。“修机场、建码头、接高铁”三件大事取得重大突破，黎湛铁路玉林至贵港段电气化改造、铁山港东岸码头实现开工，玉林民用机场、洛湛铁路玉林至梧州段电气化改造、绣江复航工程前期工作加快推进。玉铁铁路、玉铁高速公路、龙潭至山口一级公路建成通车，完成玉林至陆川、玉林至博白、玉林至兴业北市镇等二级公路改造提升，开工建设岑溪南渡至陆川、北流塘岸至新桥、贵港木格至博白水鸣二级公路。新增二级以上公路里程224.6公里，新改建农村公路3100公里，实现100%的乡镇通硬化路，100%的建制村通公路。完成水利投资54.3 亿元，病险水库除险加固401座，水电总装机达到19.2万千瓦，郁江引水等水资源配置工程建设进展顺利。建成农村人饮工程886处，完成水土流失治理548.5平方公里。

这是城乡统筹发展步伐加快、玉林大地展现新面貌的五年。我们坚持走新型城镇化道路，推动城市设施与公共服务向农村延伸，着力构建以城带乡、城乡一体的发展新格局。全市城镇化率由39.6%提高到46.5%，年均提高约1.4 个百分点。国务院批复设立福绵区。二环北路、玉北大道、玉福大道、龟山大道、二环南路、金港大道等城市主干道建成通车，市文化艺术中心等公共设施竣工投入使用，湿地公园、狮山子公园、江南绿轴、会仙河公园等生态园林建成开放，中心城区面积由56.6平方公里扩大到68平方公里，人口由55万人增加到68万人。建成国家级生态村1个，自治区级生态村143个。实施村屯绿化6614个，完成植树造林122 万亩，森林覆盖率由59.6%提高到61%。新增沼气池8.1 万户，建成农村环境连片整治示范项目179个。九洲江、南流江、北流河等重要流域污染治理有力推进，九洲江流域环境综合治理获列为国家跨地区生态补偿三个试点之一。主要污染物总量减排任务顺利完成，城乡空气环境质量和水质稳定好转，获批为国家第一批生态文明先行示范区，被评为“全国绿化模范城市”“国家森林城市”“国家园林城市”。

这是改革开放取得重大进展、经济社会焕发新活力的五年。我们全面深化经济体制、投融资、农村产权、行政审批等重点领域改革，深入实施“东靠南下、通江达海”开放战略，加快融入“一带一路”“双核驱动”战略。我市获批为全国发展改革试点城市、全国第二批农村改革试验区，成立广西首家市级农村产权交易中心，农村土地承包经营权流转率达54.5%，高于全区全国平均水平。深化行政审批制度改革，取消、下放、调整行政审批事项163项，清理规范行政许可、非行政许可事项310项。深化投融资改革，新增银行8家、非银行业金融机构44 家，实现县县建立村镇银行。全面启动商事制度改革，企业登记注册时间最快2天办结，市场主体由13.7万户发展到17.6 万户，注册资本总量突破900亿元。成功举办第八至第十一届玉博会、第三至第七届药博会、第一至第五届陶博会、第五届广西发明创造成果展览交易会，获评为全国优秀会展城市。累计引进项目2490个，到位资金2403亿元，引进中钢、华电、和黄医药、康美药业、康臣药业等一批大企业大集团。

这是各项社会事业健康发展、人民生活跃上新水平的五年。我们坚持民生优先，深入实施城乡居民收入倍增计划，加大民生保障力度，让人民群众得到更多实惠。财政民生投入达到783.9亿元，是“十一五”时期的3.3倍。连续五年实施为民办实事工程，办成一批群众热切期盼的实事好事。精准扶贫工作扎实推进，五年减少贫困人口45万人。新增城镇就业19.6万人、农村劳动力转移就业29万人，城镇登记失业率控制在4%以下。城乡居民养老保险实现全覆盖，城乡医疗保险和养老保险待遇稳步提高。发放城乡低保资金30.4亿元、救灾救助资金1.3亿元，救助群众300万人次；改造农村危房5.6万户。完成教育投入260.8亿元，年均增长18.4%；城区中小学校建设大会战有力推进，玉林高中新校区、玉州区八中、玉东小学等一批学校建成投入使用；九年义务教育巩固率由85%提高到94%，高中阶段毛入学率由69%提高到78%。公立医院改革不断深化，城乡公共卫生服务水平稳步提升。创建国家公共文化服务体系示范区扎实推进，市博物馆建成免费开放，建成城镇公共文化设施127个、村级（社区）公共服务中心798个、农家书屋1407个。完成城乡社区规范化建设160个。全民健身事业蓬勃发展，举办首届全民健身运动会、玉林市第三届运动会。城镇居民人均可支配收入由17642元增加到28842元，农民人均纯收入由5302元增加到10292元。居民消费价格总水平保持基本稳定。

这是社会管理创新不断深化、和谐稳定呈现新局面的五年。我们切实加强社会治理能力建设，促进社会公平正义，确保人民安居乐业、社会安定有序。全面完成“六五”普法任务。群众信访工作得到加强。安全生产形势持续好转。产品质量和食品药品安全监管力度不断加大。率先在全区建成未成年人教育中心和县、镇、村三级联控联防治安监控网，社会治安总体向好。国防教育和国防后备力量建设深入开展，双拥工作取得良好成效。机关事务、国资监管、外事侨务、对台事务、民族、宗教、税务、通信、无线电管理、检验检疫、农机、供销、气象、水文、消防、防震、人防、房改、住房公积金管理、档案、方志等各项工作取得新成效，妇女儿童、老龄和残疾人事业健康发展，工会、共青团、妇联等群团组织重要作用得到进一步发挥。

这是政府自身建设持续加强、管理服务提升新效能的五年。根据新形势、新任务对政府工作提出的新要求，我们切实加强民主法治建设和公务员队伍建设，积极推进行政管理制度改革，不断提高行政效能。深入开展党的群众路线教育实践活动和“三严三实”专题教育，政府机关作风进一步好转。认真执行市人大及其常委会的决议、决定，自觉接受人大、政协和社会监督，五年累计办理人大代表建议743件、政协委员提案1004件，办结率100%。稳步推进政府机构改革，调整和转变国土、环保、文化、卫生、计生、工商、质监、食品药品、市政市容等部门职能。重大决策程序机制不断完善。党风廉政建设和反腐败工作深入推进，监察和审计监督进一步加强。政务服务体系不断完善，在全区率先实现乡镇政务服务全覆盖，政务公开和信息公开持续深化。绩效管理工作不断加强，连续五年获全区一等奖。

各位代表，回顾“十二五”的工作，我们深感成绩来之不易，这是自治区党委、政府和市委正确领导的结果，是市人大及其常委会、市政协监督支持，各民主党派、工商联积极参政议政的结果，是全市干部群众抢抓机遇、团结奋斗的结果。在此，我谨代表市人民政府，向全市人民，向中央及自治区直属驻玉单位、驻玉部队、武警官兵、公安干警，向各民主党派、工商联、各人民团体和社会各界人士，向所有关心、支持玉林建设发展的港澳台同胞及海内外朋友，表示衷心的感谢和崇高的敬意！

五年来的实践使我们深刻体会到，推动玉林科学发展，必须全面贯彻创新、协调、绿色、开放、共享的发展理念，主动适应新常态、把握新常态、引领新常态，用新思路新举措推动新发展；必须坚持发展第一要务，着力提高发展质量和效益，不断推动我市发展迈上更高水平；必须加快经济结构调整和发展方式转变，推进产业转型升级，增强经济发展的动力和活力；必须深化改革开放，努力破解思想和体制束缚，不断增创发展新优势；必须加强生态建设，把生态文明建设融入经济社会建设各领域各环节，促进人与自然和谐发展；必须把改善民生作为最大责任，着力解决好关系群众切身利益的问题，使全市人民共享改革发展成果。

在总结五年发展成绩的同时，我们也清醒地看到，全市经济社会发展还面临很多困难和挑战。主要表现在：经济总量小，人均水平低，与全国全区同步全面建成小康社会任务艰巨；经济结构和产业结构不合理，传统产业增长动力减弱，新兴产业还没有形成规模，转方式调结构难度大；交通瓶颈制约突出，城乡基础设施滞后；基本公共服务和社会保障体系仍不完善，保障改善民生任务艰巨；资源环境约束趋紧，生态环境保护压力较大。这些困难和问题，我们一定高度重视，切实采取有力措施加以解决，继续把全市各项事业推向前进。

二、“十三五”时期的目标任务

“十三五”时期是全面建成小康社会的决胜阶段，是建成“两城市一中心”的攻坚阶段。综观国内外形势，我市总体上机遇大于挑战。世界经济在深度调整中曲折复苏。我国经济长期向好的基本面没有改变，新的增长动力、新的发展方式、新的增长点不断涌现。国家大力实施“一带一路”战略，加快打造中国-东盟自由贸易区升级版，支持广西构建面向东盟的国际大通道、西南中南地区开放发展新的战略支点、“一带一路”有机衔接的重要门户，自治区深入实施“双核驱动”发展战略，为我市带来了重大发展机遇。经过“十二五”的快速发展，我市步入工业化中期阶段，跨进中等收入水平，工业化、城镇化进一步提速，产业聚集效应加强，经济结构不断优化，发展动力不断增强。“十三五”时期，是我市大有可为的重要战略机遇期。

根据市委四届六次全会通过的《关于制定国民经济和社会发展第十三个五年规划的建议》，市政府组织制定了《玉林市国民经济和社会发展第十三个五年规划纲要（草案）》。纲要（草案）提出了我市“十三五”时期经济社会发展的指导思想、总体目标、主要任务和重大举措，经本次大会审议批准后，市政府将作为施政的依据，认真付诸实施。

“十三五”时期，政府工作的总体要求是：以邓小平理论、“三个代表”重要思想、科学发展观为指导，全面贯彻党的十八大和十八届三中、四中、五中全会精神，深入贯彻习近平总书记系列重要讲话精神，按照“五位一体”总体布局和“四个全面”战略布局，牢固树立创新、协调、绿色、开放、共享发展理念，大力推进“东靠南下、通江达海”开放合作，深入实施“大交通、大城市、大产业、大商贸、大田园”五大战略，加快推进“交通基础设施建设、产业转型升级、精准脱贫、社会治理能力建设”四大攻坚，着力推动经济平稳较快发展，着力提高发展质量和效益，着力增进人民福祉，把玉林建设成为广西乃至西南中南地区出海新通道、北部湾经济区开放开发新增长点、珠江—西江经济带开放合作新高地，与全国全区同步全面建成小康社会，基本建成区域性大城市、国家非公经济发展示范城市和北部湾城市群商贸中心。

“十三五”时期，我市经济社会发展的主要目标是：

——经济保持较快增长。地区生产总值年均增长8%以上，比2010年翻一番以上；财政收入年均增长7%，固定资产投资年均增长13%，工业增加值年均增长9%，社会消费品零售总额年均增长9%，外贸进出口总额年均增长6%。

——人民生活持续改善。居民人均可支配收入年均增长8%，比2010年翻一番以上；农村贫困人口全部脱贫，就业、教育、医疗、文化、社保、住房等公共服务体系更加健全。

——环境质量显著提高。主体功能区布局和生态安全屏障基本形成；节能减排降碳全面完成自治区下达任务，空气、水体、土壤环境质量优良；生态经济体系基本建成。

——改革开放全面深化。重点领域和关键环节改革取得新成果，全面深化改革完成阶段性目标；“东靠南下、通江达海”开放格局和开放型经济体系基本形成。

——民主法治全面加强。社会治理能力明显提升，群众安全感满意度全面提升，公民文明素质和社会文明程度明显提高，形成共建共享、团结进步、平安和谐的社会局面。

围绕上述目标，今后五年，重点要抓好以下八方面工作：

（一）构建大交通。持续推进“修机场、建码头、接高铁”三件大事。建成玉林民用机场，布局建设容县、龙潭通用机场。建成黎湛铁路电气化改造项目，开工洛湛铁路玉林至梧州段电气化改造项目，实现与全区高铁路网互联互通。积极争取张家界经桂林、玉林至海口高铁列入国家铁路网中长期规划。争取贺州经梧州、玉林至北海城际铁路纳入自治区规划并开工建设。确保铁山港东岸码头投入运营，绣江复航开工建设。推进玉林至湛江、玉林至荔浦、松旺至铁山港、北流清湾至南宁苏圩等高速公路建设，实现县县通高速公路。规划建设市域轨道交通，构建中心城区与各县（市、区）之间20分钟交通圈。二环西路建成通车，实现二环路大循环。规划建设三环路，完成210公里快环路前期工作。实施国道干线公路和乡村公路提级改造，推进镇镇通二级路，实现村村通硬化路目标。

（二）建设大城市。构建全域玉林规划体系。推进玉林主城区与北流城区同城化发展，加快新区开发建设，完善老城区功能配套，到2020年建成人口120万、面积150平方公里的区域性大城市。实施大县城战略和百镇扶持壮大工程，每个县（市、区）打造1-3个中心镇。提升人口城镇化水平，到2020年实现城镇新增人口90万，常住人口城镇化率达到55%。实施重大公共设施和基础设施工程，完善城乡现代配电、供水、通信、地下综合管廊等基础设施网络。完善城市治理体系，提高城市治理能力，着力解决城市病等突出问题。

（三）培育大产业。抓住“中国制造2025”全面实施的契机，大力推进工业强市，打造工业产业升级版。实施“互联网+工业”行动，加大机械制造、健康食品、有色金属、电子信息、陶瓷等产业转型升级。支持玉柴二次创业，推进玉柴智能制造，打造千亿玉柴，建设成为“一带一路”现代化机械装备制造业生产基地和出口基地。实施战略性新兴产业倍增计划，重点发展先进装备制造、新能源、新材料、节能环保、新一代信息技术、生物制药、新能源汽车等新兴产业。加快推进重点园区建设，把龙潭产业园建设成为产港城一体化的北部湾临港产业新城，玉柴产业新城成为国内先进的新型装备（内燃机）制造基地和国家火炬计划内燃机特色产业基地，中医药健康产业园成为辐射东盟和粤港澳的中医药产业集聚地，玉林高新技术产业开发区成为科技创新创业示范区，争创国家级高新技术产业园区。

（四）发展大商贸。培育壮大银丰国际中药港和中国-东盟（玉林）康美中药材（香料）交易中心、毅德国际商贸城、宏进农副产品批发市场、北流国际陶瓷贸易城、龙潭进口再生资源园区等五大专业市场，到2020年培育形成年交易额100亿元以上市场1－2个。建成1个自治区级、5个市级现代服务业集聚区。组建中国-东盟（玉林）中药材交易所，进一步打响“中国南方药都”品牌。加快玉林交通商贸物流园、空港物流园等项目建设，打造桂东快速物流分拨中心。大力发展电子商务，建成一批电子商务平台。加快“五彩田园”、大容山、玉东湖、玉西湖、中泰（玉林）国际旅游文化产业园等景区建设，打造一批国家4A级以上景区、国家旅游度假区、星级乡村旅游区和星级农家乐，创建一批特色旅游名县名镇名村，建设广西生态休闲旅游目的地和国家健康旅游示范市。

（五）打造大田园。加快发展现代农业，做大田园经济。建设广西粮食主产区和优质稻高产示范区，确保粮食播种面积稳定在470万亩以上，粮食总产稳定在180万吨以上，打造中国南方农谷。推进100个现代特色农业示范区、100个合作社示范社、100个示范家庭农场建设，到2020年新增自治区级以上农业龙头企业3－5家、超10亿元农业企业2－3家、超亿元企业10家。发展中草药规模种植，建设中国南药种植基地。加快发展生态养殖，发展水产畜牧等农产品精深加工业。建设玉林-北流-容县和九洲江生态景观带，实施“风情小镇”创建工程，建设一批田园风情小镇和村落，构建田园生态体系，打造生态田园风光，打响“五彩玉林、田园都市”品牌。加强九洲江、南流江、北流河等重点流域环境综合治理。深入推进“美丽玉林”乡村建设，加大清洁乡村、生态乡村、宜居乡村、幸福乡村建设力度。

（六）深化改革开放。深化农村综合改革，完成第二批全国农村改革试验区试验任务和农村集体经营性建设用地入市试点任务。深化国资国企、财税金融、行政管理、医药卫生、文化、教育、价格、审计等领域改革。大力推进“引金入玉”，引进各类新型金融机构，加快发展地方金融业。积极参与国际产能合作，推动机械、陶瓷、医药食品、服装皮革等优势产业走出去。深入实施加工贸易倍增计划，规划建设加工贸易集聚区。主动融入北部湾经济区同城化发展，积极参与珠江-西江经济带开放开发。深化面向东盟的开放合作。加强海峡两岸农业合作试验区建设。继续办好玉博会、药博会、陶博会，密切与侨团、商会的交往，吸引玉商、外商、侨商来玉林投资创业。实施非公有制强优企业培育计划，培育做大一批非公企业。

（七）提升社会治理能力。加强思想道德和社会诚信制度化建设，深化文明创建活动，提升社会公众责任感，在全市形成弘扬社会主义核心价值观共筑中国梦的良好氛围。全面推进社区规范化建设，推动社区实行自我管理、自我教育、自我服务、自我监督。培育和发展社会组织，支持社会组织承接就业援助、教育培训、医疗卫生、科技文体、养老助残、慈善帮困等公共服务功能。推动志愿服务制度化、规范化、常态化。加强法制宣传教育工作，健全群众利益表达、协调、保护机制，完善社会矛盾调解体系，健全社会治安立体化防控体系，全面提升群众安全感满意度。完善公共安全体系，及时排查消除安全隐患，切实抓好食品药品安全，确保人民群众生命财产安全。

（八）增进人民福祉。坚持保基本、兜底线、促公平，提高城乡基本公共服务水平，让全市人民共同迈入全面小康社会。坚决打赢脱贫攻坚战，确保到2020年贫困人口全部脱贫。大力推进就业创业，持续增加城乡居民收入。完善社会保障体系，实现应保尽保。提高住房保障水平，规划改造棚户区76个，改造棚户5.1万户。坚持教育优先发展，统筹发展各级各类教育，到2020年建成自治区示范性普通高中15所以上，自治区级以上示范性中等职业学校10所。实施“健康玉林”行动计划，提升群众健康保障水平，创建国家卫生城市。巩固提升国家公共文化服务体系示范区创建成果，促进基本公共文化服务标准化、均等化。

三、2016年的主要工作

2016年是全面建成小康社会决胜阶段的开局之年，做好今年的工作，实现“十三五”良好开局，对实现“两个建成”目标具有十分重要的意义。综合分析发展环境和自身条件，我市经济社会发展的主要预期目标为：地区生产总值增长8%-8.5%；财政收入增长7%，固定资产投资增长13%，规模以上工业增加值增长9%，社会消费品零售总额增长9%，进出口总额增长6%；居民人均可支配收入增长8%，居民消费价格涨幅控制在2.5%左右，城镇登记失业率控制在4%以内；主要污染物排放量控制在自治区下达指标内。

实现以上目标任务，要重点抓好以下十个方面工作：

（一）大力扶持实体经济发展。把实体经济健康发展作为经济增长的根本所在，优化对实体经济的扶持政策和服务举措。

积极服务实体企业。深入开展“实体经济服务年”活动，继续加大对企业的服务扶持力度，支持玉柴、中金、银亿、旺旺、燕京、南方食品、三环等龙头企业做大做强做优。开展降低企业成本行动，帮助企业正税清费，规范涉企收费，降低企业财务成本、用电成本、人工成本、物流成本、制度性交易成本，促进企业持续健康发展。实施企业上规上市激励政策，新增规模以上工业企业25家，力争培育10家企业上市。

积极推动产业集聚。树立总部经济理念，营造总部经济发展的良好环境，做大做强南达集团、兴鹏鞋业、黑五类等总部经济，吸引中金公司、诚达鞋业等企业总部落户玉林。推动城区电动车企业退城进园，实现集聚发展。推动中草药种植企业化、集团化发展，力争新组建10家中草药规模种植企业，形成中草药种植加工产业链。加快整合林产资源和矿产资源，引导规模小、效益差的企业兼并、重组，淘汰落后产能，推动资源规模化、集约化利用。抓好城区文化、体育产业的开发和经营管理。

积极化解要素制约。大力争取国家专项建设基金，支持城投公司、地产集团、交旅集团等有实力企业发行债券，组建产业项目基金，多渠道筹措发展资金。加强基础设施和公共事业特许经营权管理，推进政府和社会资本合作。整合机关、企事业单位的土地和资金等资源，盘活闲置资产。创新土地收储出让机制，实行土地收储与安置区建设责任挂钩，安置区与项目同步建设，解决征地拆迁难问题。

（二）大力推进工业转型升级。工业是调结构、转方式的主战场，更是稳增长的强大引擎。坚持以创新驱动为引领，推动工业结构调整，加快工业转型升级步伐。

加强创新能力建设。加快中小企业科技创新孵化平台建设，力争建成自治区级玉林高新区，新增孵化器1000平方米，新增入孵企业20家。加快建设国家内燃机及零部件产品质量监督检验中心、广西电动车产品质量监督检验中心。健全产学研合作机制，鼓励企业加大研发投入。新增自治区级以上高新技术企业5家、市级工程技术研究中心12家。深入实施质量兴市战略，引导和激励各行各业不断提高质量水平。深入实施人才强市战略，进一步激励科技人才创新创业。

做强做优传统产业。启动新一轮技术改造升级工程，积极培育新产品，推动黑五类、五丰机械等企业新产品产业化；大力引进工业机器人、精密数控机床生产研发企业，提高机械制造、健康食品、陶瓷、有色金属等产业的产品技术、工艺装备、能效环保水平。支持玉柴加快柴油机关键零部件制造与开发、气体发动机二期等项目建设，推动国V车用柴油机全面量产。加快中金公司热轧冷轧项目建设，大力引进不锈钢深加工企业，力争不锈钢产业产值超100亿元。加快康美药业等10个中医药产业项目建设，力争中药提取基地年处理中药材能力40万吨以上。完成工业投资810亿元、技改投资590亿元。

培育壮大新兴产业。加大政策支持和招商引资力度，大力培育发展新兴产业。引进稀土、石墨烯、碳酸钙等材料精深加工企业，推动龙潭稀土永磁发电机、龙潭高分子塑料合金、中铝新材料产业园、兴业碳酸钙产业园等项目建设，加快发展新材料产业。推进华电热电联产项目建设，积极引进新能源企业，推进一批风电项目，加快发展新能源产业。加大利维重工、米勒电动汽车等企业培育力度，发展新能源汽车产业。推进龙潭产业园废旧船舶拆解、年产30万吨再生铜冶炼及综合回收利用、龙潭进口再生资源园区“城市矿产”示范基地建设，加快发展再生资源产业。大力培育生物制药、节能环保、新一代信息技术等新兴产业。

加强工业园区建设。创新园区投资模式，搭建园区投融资平台，增强园区发展能力。以龙潭产业园、玉柴产业新城、中医药健康产业园等八大园区为重点，大力推进园区基础设施和重点项目建设。完成园区基础设施建设投资23亿元以上。重点推进一批重大项目建设，康臣·玉药整体搬迁入园、大参林饮片加工、银亿含镍铬固体废料无害化处理等总投资80亿元的45个项目开工建设，中金与宝钢合作不锈钢项目、玉东训达油脂花生油、玉柴中原内配年产500万只气缸套等总投资70亿元的50个项目竣工投产。园区工业总产值增长20%以上。

（三）大力推动服务业集聚发展。坚持扩大总量与优化结构并重，推进加快发展服务业行动，构建现代服务业体系。

加快现代服务业集聚区建设。强化现代服务业集聚区培育扶持，加快推进玉林市现代服务业物流集聚区、电子商务快递物流园、中国—东盟（玉林）中药材交易市场、福绵服装商贸基地、综合商品贸易集聚区、会展商务集聚区、国际健康城等7个列入自治区规划的集聚区建设，促进服务业集聚发展、增量提质。

加快商贸物流业发展。培育壮大银丰国际中药港等五大专业市场，加快新型专业市场集聚发展。积极推进万达广场、金城广场、交通商贸物流园、无水港一期等项目，加快中药港、毅德、宏进等电子结算平台建设，促进商贸物流业提质升级。新建、改扩建一批农贸市场，优化城乡市场布局。

加快特色旅游业发展。抓好大容山、“五彩田园”、真武阁、世客城、六万大山、鹿峰山、宴石山等重大旅游项目建设，提高景区档次和品位。建设智慧旅游体系，提升旅游业服务质量。支持容县、北流创建广西特色旅游名县，创建一批特色旅游名镇名村。力争创建A级景区、星级乡村旅游区和星级农家乐20家以上，旅游总收入增长18%以上。

加快培育新型服务业态。实施“互联网+”计划，大力引进和培育电商及关联产业。创新商业模式和服务业态，加快发展电子商务、金融、云计算、物联网、工业设计、文化创意、商务会展等生产性服务业。推进生活性服务业向精细化、高品质转变，加快发展健康养老、商贸餐饮、家政服务、文体娱乐、教育培训等生活性服务业。

（四）大力促进农业提质增效。加快转变农业发展方式，积极调整农业产业结构，提升农业质量效益和竞争力，走产出高效、产品安全、资源节约、环境友好的现代农业发展道路。

抓好现代特色农业示范区建设。以示范区建设为抓手，加快推进农业转型升级。加快隆平高科、中国南药园等项目建设，促进“五彩田园”提档升级。抓好市级和各县（市、区）现代特色农业示范区建设，力争建成7个市级、20个县级、30个镇级现代特色农业示范区，全市示范区经营总收入达8亿元。

培育壮大新型农业经营主体。做好土地经营权流转工作，鼓励支持土地股份合作、土地信托等现代经营模式，发展多种形式的适度规模经营。加大家庭农场、农民合作社、农业龙头企业培育力度，每个县（市、区）培育10-20个农民合作社示范社、示范性家庭农场。培育一批新型职业农民。扶持壮大宏邦、参皇、春茂、温氏、绿野等农业龙头企业。

发展特色农业产业。切实保护耕地，严格落实粮食安全责任制，发展壮大粮食产业。按照“一村一品”“一镇一业”布局，大力发展水果、蔬菜、中药材、食用菌、生猪、家禽、水产品等特色高效农业。推进九洲江流域中药材专属区建设。加快发展林下经济，培育休闲农业、观光农业、生态农业等新型农业业态。

打造品牌农业。围绕沙田柚、铁皮石斛、香蒜、陆川猪、牛巴、桂圆肉、沉香、橘红、菠萝蜜等特色农产品，着力改良品种品质，延长农产品产业链。加大农业品牌和“三品一标”认证支持力度，提升农业品牌知名度和市场竞争力，新增认证“三品一标”产品10个以上。鼓励和支持加工企业向精深加工发展，重点推进生猪、家禽、皮革、罗非鱼、百香果等优势产品精深加工，提高附加值。加快农业标准化进程，推进农产品质量追溯体系建设，创建一批蔬菜、水果等园艺作物标准园。

（五）大力提升城市功能品位。加快转变城市发展方式，突出规划引领，强化基础支撑，加强城市管理，推动城市持续健康发展。

突出城市扩容。抓好城市总体规划修编，推进城市规划“多规合一”，统筹好国土空间、产业布局、人口规模和环境容量。加快玉东新区开发建设，推动玉州老城区改造升级，提升福绵区配套设施，促进玉北同城化发展，做大做强中心城区。加快龙港新区规划建设。继续实施大县城战略，推进博白撤县改市工作。支持重点镇和中心镇建设发展。

突出城市提质。加快推进玉东湖等重大项目，建好园博园。推进二环西路、国道324线玉林绕城路等城市干道建设，开工建设城北、城东、城南三个汽车客运综合枢纽站，启动玉林汽车站、东郊站搬迁。开展城市地下综合管廊试点，推进城市电网、通信网架空线入地改造工程。加强排污、垃圾处理、公厕改造、公共停车场等设施建设，完善城市功能。加快火车站站前广场片区、南流江中段等棚户区改造，推进老旧住宅小区、城中村综合整治。加快创建智慧城市、海绵城市，启动创建文明城市和卫生城市，提高城市品位。

突出城市管理。加大城市管理投入，推行城市管理目标责任制，建立综合、快速、高效的城市综合执法体制。建立完善数字化城市管理平台。积极推进城市管理进社区。深化“五乱”整治和“四车”治理，推进规范管理向城中村、城乡结合部、乡村延伸。依法整治违章违法建筑，大力打击公共场所非法收取停车费行为，提升城市形象，改善城市面貌。

突出城乡统筹。探索城乡一体化发展体制机制，推动城乡要素平等交换和公共资源均衡配置。加快户籍制度改革和居住证制度双落地，建立健全“人地钱”三挂钩机制，让农民工尽快享受基本均等的公共服务，实现住房、入学有保障。继续推进城乡客运一体化。

（六）大力挖掘经济增长潜力。统筹发挥投资、消费、出口“三驾马车”协调拉动作用，促进经济行稳致远。

努力扩大有效投资。优化投资结构，引导投资向重点行业、关键领域和薄弱环节倾斜。强化重大项目带动，推进235个重点项目建设，实现年度投资1562亿元。继续抓好中央投资项目和自治区层面统筹推进项目建设，确保在建和续建项目如期完成。突出抓好重大项目前期工作，做好“十三五”821个重大项目规划工作，储备一批事关全局和长远的重大项目。千方百计破解项目土地、资金等瓶颈制约。

努力促进城乡消费。积极推进专业市场提升发展，加快市旅游文化创业大街等商贸功能区、特色商业街区建设，为城乡居民提供便利、多样、规范的消费服务。开展多种形式的展销会、交易会、购物节，活跃城乡消费市场。多渠道促进城乡居民增收，重点提高中低收入人群收入，让群众能消费、敢消费、愿消费。

努力增加对外贸易。提升贸易便利化水平，积极推进通关便利化。深入实施加工贸易倍增计划，扩大进出口主体。实施外贸品牌发展战略，扶持壮大玉柴、中金、银亿、龙潭进口再生资源、三环集团等重点进出口企业，引导外贸企业转型升级。加强机械、电子、有色金属、日用陶瓷、编织工艺品、皮革等重点行业的跟踪服务，调整优化外贸结构，扩大外贸出口。

（七）大力加强基础设施建设。以“修机场、建码头、接高铁”为重点，推动交通、能源、水利等一批重大基础设施建设，全面增强发展支撑能力。

抓好交通基础设施建设。按照建设区域性交通枢纽要求，推进20个重大交通项目建设。实现玉林铁路地区枢纽扩能改造、火车站站前广场改造、黎湛铁路电气化改造等项目竣工试运行通动车，苗园路铁路涵洞改造、324国道容县至兴业段大修工程、贵港木格至博白水鸣公路、北流至新桥公路、北流城区至大容山二级公路等在建项目竣工通车。开工建设玉林民用机场及机场公路、洛湛铁路玉林至梧州段电气化改造、铁山港东岸10万吨码头及进港大道、玉林至湛江高速公路、荔浦至玉林高速公路、松旺至铁山港高速公路、岑玉线北流甘村至新圩段公路改扩建、玉林至陆川城区公路改扩建、北流文成至六靖二级路等项目。加快岑溪南渡至陆川二级公路等项目建设。全力做好绣江复航、张家界经桂林玉林至海口高铁、南宁苏圩至北流清湾高速公路等项目前期工作。协调推进贺州经梧州、玉林至北海城际铁路前期工作。

抓好能源基础建设。建成玉林－北流天然气接气工程、容县杨村风电二期等项目。加快博白马子岭风电、云飞嶂风电、四方嶂风电、大容山抽水蓄能发电等项目建设，为经济社会发展提供充足的电力保障。

抓好水利设施建设。以重大水利、民生水利、农田水利为重点，全面提升水利的支撑和保障能力。确保龙潭伟业供水工程二期、罗田水库供水工程、郁江引水管线及城北水厂扩建工程竣工运行。完成龙云水资源配置工程、蕉林水库、黄章水库、秦镜水库等项目前期工作，争取尽快开工建设。加快病险水库除险加固和防洪工程建设，推进南流江、北流河干流防洪工程，抓好小农水重点县建设，解决农田灌溉“最后一公里”问题。

（八）大力深化改革开放合作。坚持以改革开放统揽发展全局，向改革开放要活力促发展，再创经济发展新红利。

着力推进供给侧结构性改革。加快推进产业重组、“僵尸”企业处置，加大淘汰落后产能力度，进一步盘活各种闲置资源，优化资源配置，有针对性地改善和创新供给，加快形成新主体、培育新动力、发展新产业。认真落实国家化解房地产库存要求，扩大有效需求，打通供需通道，消化库存，稳定房地产市场。积极增加有效投资，补齐软硬基础设施短板，推动产业向中高端迈进，促进企业提供更高品质的产品和服务，努力增加有效供给，满足人们更好的物质精神追求。

着力深化重点领域改革攻坚。稳步推进农村改革试验区和北流农村集体经营性建设用地入市改革，加快完善农村产权流转交易市场体系，推动各类农村产权入市交易。深化投融资体制改革，积极开展股权融资、产权交易融资、债券融资等多样化融资模式。推广运用政府和社会资本合作PPP模式，加快实施31个入库项目。深化金融改革，稳步发展小额贷款公司、融资性担保公司，培育设立各类投资基金，加大金融支持实体经济力度。推进社会信用体系建设。创新完善非公有制经济发展体制机制，推动大众创业、万众创新。统筹推进经济体制、工商登记、行政审批、财税、价格机制、国资国企、国有林场、集体林权等重点领域改革，激发市场活力和社会创造力。

着力推进全方位开放合作。主动融入国家“一带一路”和自治区“双核驱动”发展战略，坚定不移实施“东靠南下、通江达海”战略，重点抓好龙港新区开发建设，实现龙潭进口再生资源园区验收开园，大力推进10个列入西江经济带基础设施建设大会战项目。举办好第16届国际传统药物学大会和第八届药博会。加强国际友好城市平台建设，深化与粤港澳和东盟国家的合作，加快推进中泰（玉林）国际旅游文化产业园、中国（玉林）-文莱中医药健康产业园等中外合作项目。加大招商引资力度，围绕重点园区、先进制造、生物制药、节能环保、商贸物流、文化旅游、现代农业、城镇化建设和交通、能源、水利基础设施建设等重点领域，着力引进一批重大项目。

（九）大力推进美丽玉林建设。顺应人民群众对良好生态环境的期待，坚持以生态为重，巩固生态优势，做好环境保护“加减法”，促进经济与生态协调发展。

强化生态城乡建设。深入开展“美丽玉林·生态乡村”活动，实施“六个一”工程，努力打造一批生态示范镇，力争完成8361个村屯绿化任务，完成植树造林20万亩。突出岭南生态特色，抓好城市绿带、绿轴、绿核建设，启动建设玉北容生态景观长廊、九洲江生态景观带，建设一批集休闲娱乐、度假观光于一体的生态旅游基地，加快构建“一区两路三江”生态格局。

强化生态基础设施建设。推进玉林（福绵）节能环保产业园建设。加快完善城乡污水处理设施，改造县级以上老城区污水管网，完成生活污水直排口截污工作；启动23个乡镇污水处理厂项目，建成20个；率先在陆川县建设一批村级污水处理设施，支持陆川县创建全国生活污水处理示范县。加快生活垃圾分类收集和处理设施建设，开工建设生活垃圾焚烧发电厂。加强农村供水设施、大型沼气池和林业生态基础设施建设。

强化生态环境治理。加大九洲江、南流江、北流河流域环境治理力度，清拆主干流200米范围内养殖场，整治禁养区外规模养殖污染企业，抓好病死畜禽、养殖废弃物无害化处理厂和有机化肥厂建设，推动沿岸工业企业搬迁入园，确保九洲江和南流江跨界断面水质稳定达标。推进规模养殖场标准化建设，大力推广高架网床等生态养殖模式。开展以治水、治气、治渣、治尘为重点的环保专项行动，加大对水泥等重点污染行业治理力度。

强化节能减排工作。加强能耗监管，继续淘汰落后产能。强化公共机构节能。加大主要污染物减排力度。抓好机动车尾气污染监管，加快淘汰黄标车和老旧车。推进城区空气自动站建设和PM10、PM2.5监控。大力发展生态产业和循环经济，加快“城市矿产”示范基地建设，打造国家再生资源回收利用体系建设试点城市。推进生态修复工程，加强重点生态功能区保护和管理。

（十）大力抓好民生事业发展。坚持发展为民，着力解决好群众最关心、最直接、最现实的利益问题，让人民群众有更多获得感。

扎实推进精准扶贫攻坚。精准实施基础设施建设、产业发展、教育培训、金融服务、公共服务保障、社会帮扶“六大扶贫攻坚工程”，确保实现贫困人口脱贫10万人，一批贫困村脱贫出列。集中力量解决革命老区、贫困村通村通屯道路、公共服务设施建设、饮水安全、农村危房改造等问题，抓好革命老区和贫困村劳动力培训。

扎实推进教育事业发展。发展学前教育，鼓励普惠性幼儿园发展。促进义务教育均衡发展，改扩建农村中小学校800所。加快普及高中阶段教育，探索多元化、集团化办学模式。大力发展现代职业教育，规划建设职业教育园区。继续推进城区中小学校建设大会战，加快玉林师院附中新校区、玉东实验小学、玉州区金港小学、玉州区秀石路小学等学校建设。实施教育惠民工程，资助学生30万人次以上。抓好广西医科大学玉林校区建设，支持玉林师范学院向综合性大学发展，提升高等教育发展水平。

扎实提升公共就业服务水平。加快创业孵化基地和农民工创业园建设，推进小额担保贷款和农民工创业担保贷款，落实就业创业扶持补贴，开展就业服务专项活动。加快基层就业和社保服务中心建设，推进农村劳动力职业技能精准培训。力争新增城镇就业3万人、农村劳动力转移就业5万人。

扎实推进文化体育事业发展。加快市图书馆新馆、档案馆新馆、群艺馆新馆、王力博物馆、非物质文化遗产展示中心、科技馆、工人文化宫、妇女儿童活动中心、青少年活动中心等公共文化设施建设，提升基本公共文化服务水平，确保成功创建国家公共文化服务体系示范区。新建100个村级公共服务中心。重视文物、非物质文化遗产保护。实施自然村通有线广播电视、无线宽带工程。发展全民健身、竞技体育和体育产业，举办好第二届全民健身运动会。

扎实推进卫生计生事业发展。深化医药卫生体制改革。抓好公立医院改革。加快推进县乡医疗服务一体化，提升社区卫生服务能力。加大卫生基础设施投入力度，改善居民就医环境，提高医疗诊治水平和服务能力。加快市骨科医院整体搬迁项目、市中心医院、儿童医院建设。落实好全面两孩政策。

扎实推进社会保障体系建设。推进社保扩面征缴，深化机关事业单位养老保险制度改革。落实北部湾基本医疗保险和生育保险同城化政策，加强基本医疗保险付费总额控制。推进全民参保登记计划、城镇居民大病保险、社保服务标准化建设三个国家级试点，开展社会保障“一卡通”制发换卡工作。

扎实推进社会治理创新。加快城乡社区规范化建设，积极推进网格化管理和精细化服务。健全社会矛盾纠纷预防化解机制，加强信访网络信息平台建设，加大信访积案化解力度，努力把矛盾纠纷化解在基层、解决在萌芽状态。加强法制宣传教育，构建覆盖城乡的公共法律服务体系。健全立体化治安防控体系，完成中心城区“天网工程”和全市乡镇高清视频监控系统建设。严密防范和严厉打击违法犯罪活动，强化反恐维稳工作，不断提升人民群众的安全感满意度。加强应急管理能力建设，提高保障公共安全和应对突发事件能力。抓好安全生产、食品药品安全等工作。

扎实推进为民办实事工程。重点抓好社保、健康、教育、强基、安居、土地整治、农补、文化、生态、交通等民生项目，完成80个自然村道路硬化，建成1万套城镇保障性住房、18所学校、417个自治区级绿化示范村屯。

加强国防动员和后备力量建设，支持军队改革和驻玉部队建设，广泛开展双拥共建活动，推动军民融合发展。落实民族宗教政策，促进民族团结和宗教和谐。依法保障妇女、未成年人和残疾人的合法权益。做好老龄工作，加快社会养老服务体系建设。加强全民科学素质和道德素质工作。充分发挥工会、共青团、妇联等群团组织的重要作用。继续做好统计、外事、侨务、对台事务、人防、气象、防震、水文、测绘、无线电管理、方志、档案等工作。

各位代表，做好新形势、新常态下的政府工作，必须切实加强政府自身建设。我们将认真实施中共中央、国务院《法治政府建设实施纲要（2015–2020年）》，深入推进依法行政，严格依照法定权限和程序行使权力、履行职责，强化严格执法，加快建设法治政府。严格执行市人大及其常委会的决议、决定，定期向人大及其常委会报告工作，自觉接受人大及其常委会监督，积极支持人民政协履行政治协商、民主监督、参政议政职责，切实做好人大代表建议、批评和意见及政协委员提案办理工作。巩固“三严三实”专题教育成果，形成作风建设长效机制，加强公务员队伍建设，强化政府绩效管理，完善行政问责机制，深化行政审批制度改革，全面推行政务公开和政府信息公开，不断提高政府执行力和公信力。严格落实党风廉政建设“两个责任”，编织科学严密的制度“笼子”，加强审计监督和行政监察工作，坚决查处发生在群众身边的“四风”和腐败问题，树立为民务实清廉的政府形象。

各位代表！梦想承载希望，实干开创未来。让我们在市委的坚强领导下，紧紧团结和依靠全市人民，进一步解放思想，同心同德，真抓实干，全面开创玉林经济社会发展新局面，为实现“两个建成”目标而努力奋斗！
